# 폐경 여성의 삶의 질 영향요인

**DOI:** 10.4069/kjwhn.2020.11.14

**Published:** 2020-12-07

**Authors:** Hyunsook Shin, Eunjoo Lee

**Affiliations:** 1 Yein Woman Clinic, Changwon, Korea; 1 예인여성병원; 2 Department of Nursing, Kyungnam University, Changwon, Korea; 2 경남대학교 간호학과

**Keywords:** Hormone replacement therapy, Menopause, Quality of life, Women, 호르몬 대체요법, 폐경, 삶의 질, 여성

## Abstract

**Purpose:**

This study aimed to identify factors influencing quality of life in post-menopausal women.

**Methods:**

The participants were 194 post-menopausal women who visited a women’s clinic in Changwon, Korea from July 1 to August 31, 2018, and completed questionnaires containing items on menopausal symptoms, marital intimacy, current menopausal hormone therapy (MHT), and quality of life. Collected data were analyzed by descriptive statistics, the independent t-test, Pearson correlation coefficients, and multiple regression using SPSS for Windows version 23.0.

**Results:**

Quality of life had a significant negative correlation with menopausal symptoms (r=–.40, *p*<.001), and a significant positive correlation with marital intimacy (r=.54, *p*<.001). The factors influencing the quality of life of post-menopausal women were current MHT (t=6.32, *p*<.001), marital intimacy (t=4.94, *p*<.001), monthly family income (t=4.78, *p*<.001), menopausal symptoms (t=–4.37, *p*<.001), and education level (t=3.66, *p*<.001). These variables had an explanatory power of 59.2% for quality of life in post-menopausal women.

**Conclusion:**

In order to improve the quality of life of post-menopausal women, nursing interventions are needed to help menopausal women choose appropriate MHT, alleviate menopausal symptoms, and increase marital intimacy. Interventions should also be prioritized for women of a low educational level and with a low income in consideration of their health problems.

## Introduction

### 연구 필요성

폐경은 난소 기능의 노화에 의해 에스트로겐의 분비가 감소하고 배란과 월경이 완전히 중단되는 현상을 말한다[1]. 여성의 생애주기에서 정상적인 과정이지만 폐경으로 인한 증상이나 질환들은 여성의 일상생활을 저해하고 삶의 질을 떨어뜨릴 수 있다[2]. 우리나라 여성의 평균 폐경 연령은 48–55세이며[1], 최근 여성의 기대수명이 85.7세로[3] 길어짐에 따라 폐경 이후 삶과 건강에 대해 관심이 높아졌다.

삶의 질(quality of life)은 삶의 위치와 삶의 가치 체계, 목표, 기대, 기준 및 관심사와 관련된 삶에 대한 개인의 인식으로[4], 신체적, 심리적, 사회적 및 환경 전반에 걸친 포괄적인 개념이다. 폐경 여성의 삶의 질은 폐경으로 인한 신체적∙정신적 변화와 더불어 노화의 진행으로 인해 부정적인 영향을 받게 된다[5]. 폐경으로 인한 변화는 40대 중∙후반에서 시작되어 점차 진행되는데[2], 폐경 여성들은 폐경 전 여성들에 비해 체중, 체질량지수, 허리둘레, 체지방량, 혈당, 중성지방, 인슐린 등이 증가하며, 특히 고콜레스테롤혈증 발생 위험이 증가한다[2]. 치료하지 않은 경우 고혈압, 골다공증, 심혈관질환, 우울 등 일상생활에 지장을 줄 수 있으며, 특히 비뇨생식기 증상은 배우자와의 관계나 삶의 질을 떨어뜨릴 수 있다[6,7]. 따라서 폐경 여성의 삶의 질과 관련된 요인들이 어떠한 것인지를 확인하고 이를 중재하는 것은 의미 있는 일일 것이다.

폐경 증상은 폐경 여성의 삶에 질에 영향을 미치는 요인으로 보고되고 있다[7]. 흔한 증상으로는 수면장애, 우울한 감정, 기분 변화, 기억력 및 집중력 감소, 자신감과 의욕 저하, 과민성, 성욕 저하, 근육 통증 등이 있다[6]. 이러한 증상들은 일상생활에 지장을 주거나 건강상태를 악화시킬 수 있으며[6], 정서적 불안정과 우울과 같은 정동장애로 이어지기도 한다[8,9]. 혈관운동 증상의 경우 폐경 여성의 대부분이 경험하며, 특히 열성 홍조는 폐경 여성의 삶에 가장 영향을 미치는 증상으로, 불쾌한 발열감, 적색 피부변화, 발한과 맥박 상승을 동반하며 심할 경우 오한, 심계항진, 현기증이 발생할 수도 있다[9]. 폐경과 관련된 혈관운동 증상, 신체적 증상, 심리사회적 증상, 비뇨생식기 증상 등에 관해 예방적 차원에서 초기부터 관리할 필요성이 있다[2]. 따라서 폐경 증상에 대해 경각심을 가지고 조기에 적극적으로 관리한다면 폐경 증상의 감소와 함께 합병증을 예방하여 건강한 노년의 삶을 유지할 수 있을 것이다.

부부친밀도는 부부 관계에서 가장 필요로 하는 관계성을 나타내는 개념으로, 중년 여성의 삶의 질을 향상시키는 데 중요한 역할을 하는 변수이다[10]. 폐경 여성은 가족의 변화로 인한 상실감, 노화현상 및 폐경 증상 등으로 정신적 위기를 경험하지만, 부부친밀도가 높은 경우 삶의 질이 향상될 수 있다[10]. 폐경 이후 여성은 폐경 이행기 여성에 비해 부부친밀도가 상대적으로 낮은 편인데, 이는 폐경이 배우자와의 관계에 부정적 영향을 미쳤기 때문이다[11]. 특히 폐경 증상이 심할 경우 부부친밀도가 낮아졌으며, 신체적, 정신적 건강 관련 삶의 질도 낮게 나타났다[7]. 폐경 여성의 삶의 질에 대한 포괄적인 접근을 위해 배우자와의 관계적 측면뿐 아니라 성 활동이나 성 표현을 포함한 부부친밀도와의 관계에 대해 살펴볼 필요가 있겠다.

한편, 폐경기 호르몬요법은 폐경 여성의 혈관운동계와 비뇨생식기 증상을 치료하는 데 효과적인 것으로 알려져 있으며, 특히 60세 이하나 폐경 발생 10년 이내의 갱년기 증상에서 사용할 경우 최대의 효과와 안정성이 보장된다[12]. 수술로 인한 조기난소부전 등과 같은 인공폐경이 자연폐경에 비해 열성 홍조와 같은 증상이 더 자주, 보다 심하게 나타나기 때문에 호르몬요법이 고려된다[13]. 조기 에스트로겐 감소에서는 심혈관질환, 골다공증, 성기능 감소, 비뇨생식기 증후군 등의 위험을 방지하기 위해 최소 폐경 연령인 52세까지 치료가 권유된다[12]. 그러나 일부 폐경 여성들은 폐경기 호르몬요법을 결정할 때 암 발생과 같은 부작용의 위험을 이유로 높은 수준의 갈등을 경험하는 것으로 나타났다[14]. 폐경기 호르몬요법은 개인의 증상, 위험도, 치료 목적에 따라 달라질 수 있으며, 충분한 상담을 통해 적절한 호르몬제를 사용한다면 폐경으로 인한 증상을 줄이고 나아가 여성의 삶의 질 향상도 기대할 수 있을 것이다. 따라서 폐경기 호르몬요법 여부에 따라 폐경 여성의 삶의 질이 어떠한 영향을 받는지에 대해 명확하게 확인해 볼 필요가 있다.

이외에도 연령, 결혼상태, 한 달 가족수입, 교육수준, 초경 연령, 폐경 연령[5], 폐경 후 기간, 만성질환 유무, 음주 여부[7] 등과 같은 변인과 폐경 여성의 삶의 질과의 관련성에 관하여 확인할 필요가 있다.

폐경 여성의 삶의 질에 관한 국내외 선행연구를 살펴보면, 폐경 증상[5,15,16], 극복력, 희망, 부부친밀감, 가족 지지[10], 성기능[17], 우울증상, 부부친밀도[7], 폐경 형태와 대사증후군 위험[18] 등이 있었으나 부부친밀도, 폐경 증상, 삶의 질, 그리고 폐경기 호르몬요법을 함께 살펴본 연구는 드물었다. 이에 본 연구는 폐경 여성의 부부친밀도, 폐경 증상, 폐경기 호르몬요법이 삶의 질에 어떠한 영향을 미치는지 확인하고, 이를 근간으로 폐경 여성의 삶의 질 향상을 위한 간호중재 프로그램의 기초자료를 제공하고자 한다.

### 연구 목적

본 연구의 목적은 폐경 여성의 삶의 질 영향요인을 파악하는 것으로, 구체적인 목적은 다음과 같다.

• 폐경 여성의 삶의 질, 폐경 증상, 부부친밀도, 폐경기 호르몬요법 실태를 파악한다.

• 폐경 여성의 인구사회학적 특성에 따른 삶의 질 차이를 파악한다.

• 폐경 증상, 부부친밀도, 삶의 질 간의 관계를 파악한다.

• 폐경 여성의 삶의 질 영향요인을 파악한다.

## Methods

Ethics statement: This study was approved by the Institutional Review Board of Kyungnam University (1040460-A-2018-030). Informed consent was obtained from the participants.

### 연구 설계

본 연구는 폐경 여성의 삶의 질 영향요인을 파악하기 위한 상관성 조사연구이다.

### 연구 대상

본 연구의 대상은 경상남도 창원시 Y여성전문병원의 외래를 내원한 폐경 여성을 편의 표집하였으며, 구체적인 선정기준은 다음과 같다. (1) 폐경 후 1년이 경과하여 의사로부터 폐경을 확인 받은 여성, (2) 본 연구 목적을 이해하고 연구 참여에 자발적으로 동의한 여성.

연구 대상자 수는 G power 3.2 프로그램을 사용하였고, 선행연구[17]를 참고하여 회귀분석에 필요한 유의수준(alpha) .05, 검정력(power) .95, 효과크기(effect size) .15, 예측요인 11개로 산출하였으며, 그 결과 최소 178명이 요구되었다. 본 연구에서는 탈락률 10%를 고려하여 200명에게 설문지를 배부하였으며, 이 중 불성실하게 응답한 4명과 인공폐경 2명을 제외한 194명이 최종 연구 대상이었다.

### 연구 도구

#### 삶의 질

삶의 질은 세계보건기구(World Health Organization, WHO)에서 개발한 WHO 삶의 질 척도(WHO Quality of Life, WHOQOL) [4]를 Min 등 [19]이 한국형으로 수정·보완한 한국어판 WHOQOL 척도를 사용하여 측정하였으며, 도구 사용에 대해 저자에게 허락을 받았다. 이 도구는 전반적인 삶의 질 2문항(1, 2), 신체적 건강 영역 7문항(3, 4, 10, 15–18), 심리적 영역 6문항(5–7, 11, 19, 26), 사회적 관계 영역 3문항(20, 21, 22) 및 환경 영역 8문항(8, 9, 12–14, 23–25)의 총 26문항으로 구성되어 있다. 각 문항은 ‘전혀 아니다’ 1점에서 ‘매우 그렇다’ 5점의 5점 Likert 척도이며, 이 중 3번, 4번, 26번 문항은 역환산하였다. 최저 26점에서 최고 130점으로 점수가 높을수록 삶의 질이 높음을 의미한다. Min 등[19]의 연구에서 도구의 신뢰도(Cronbach’s α)는 .89였으며, 본 연구에서는 .94였다.

#### 폐경 증상

폐경 증상은 Sarrel [20]이 개발한 폐경 증상 도구(Menopause Symptom Index)를 Jo와 Lee [21]가 한국인에 맞게 수정·보완한 도구를 사용하여 측정하였으며, 도구 사용에 대해 저자에게 허락을 받았다. 이 도구는 신체적 증상 11문항(1–11), 정신적 증상 4문항(12–15), 성적 증상 5문항(16–20)의 총 20문항으로 구성되어 있다. 각 문항은 ‘없다’ 0점, ‘가끔’ 1점, ‘자주’ 2점의 3점 Likert 척도로, 최저 0점에서 최고 40점으로 점수가 높을수록 폐경 증상이 심한 것을 의미한다. Jo와 Lee [21]의 연구에서 도구의 신뢰도(Cronbach’s α)는 .76이었으며, 본 연구에서는 .89였다.

#### 부부친밀도

부부친밀도는 Waring과 Reddon [22]이 개발한 부부친밀도 질문지(Marital Intimacy Questionnaire)를 Kim [23]이 수정·보완한 도구를 사용하여 측정하였으며, 도구 사용에 대해 저자에게 허락을 받았다. 이 도구는 부부 간 의사소통 1문항(1), 상호의존도 1문항(2), 여가활동 1문항(3), 성생활 만족 1문항(4), 감정 표현 2문항(5, 7), 가족관계 유지 1문항(6), 결혼 생활의 안정감 1문항(8)의 총 8문항으로 구성되어 있다. 각 문항은 ‘전혀 그렇지 않다’ 1점에서 ‘매우 그렇다’ 4점의 4점 Likert 척도로, 최저 8점에서 최고 32점으로 점수가 높을수록 부부친밀도가 높음을 의미한다. Kim [23]의 연구에서 도구의 신뢰도(Cronbach’s α)는 .92였으며, 본 연구에서는 .90이었다.

#### 폐경기 호르몬요법 실태

폐경기 호르몬요법 실태는 선행연구[24,25]를 참조하여 호르몬제 사용 시작 연령, 투여 기간, 선택 이유, 투여 중 정기검진 기간, 치료 효과, 완화된 증상, 부작용 설명을 들은 경험, 부작용을 설명한 사람, 부작용 경험 유무, 부작용 증상, 사용 중단 경험 유무, 중단 이유, 재사용 이유를 포함하여 총 13문항으로 구성되었다.

본 연구에서 폐경기 호르몬요법은 경구 호르몬을 의미하며, 경구용 에스트로겐과 프로게스테론 병합, 혹은 경구 에스트로겐 단독 복용 방법을 말한다[26].

#### 인구사회학적 특성

인구사회학적 특성은 연령, 교육수준, 한 달 가족 수입, 현재 흡연 여부, 현재 음주 여부(주 1회 이상이면 음주에 해당), 내과적 질환 유무, 직업 유무, 폐경 후 기간, 현재 폐경기 호르몬요법 여부 등 총 9문항으로 구성되었다. 내과적 질환은 고혈압, 당뇨, 이상지질혈증, 골다공증 등의 질환을 포함한다.

### 자료 수집

본 연구의 자료 수집은 2018년 7월 1일부터 2018년 8월 31일까지 시행하였다. 창원시에 소재한 Y여성전문병원의 병원장에게 연구에 대한 허락을 얻은 뒤 연구자가 직접 자료 수집을 진행하였다. 해당 병원의 외래를 방문한 폐경 여성들에게 연구의 목적, 방법, 비밀 보장, 익명성, 그리고 연구 참여를 하지 않거나 참여 도중 중단하더라도 어떠한 불이익도 없음을 설명하였다. 연구에 자발적인 참여 의사를 밝힌 대상자에게 대상자 선정기준에 부합되는지 질문하고, 적합한 경우 연구 참여 동의서를 서면으로 작성한 후 설문을 실시하였다. 설문 작성에 소요되는 시간은 10–15분이었으며, 작성 후 곧바로 회수하였고, 소정의 답례품을 지급하였다.

### 자료 분석

수집된 자료는 IBM SPSS for Win ver. 23.0 통계 프로그램(IBM Corp., Armonk, NY, USA)을 사용하여, 대상자의 인구사회학적 특성, 폐경 증상, 부부친밀도, 폐경기 호르몬요법 실태 및 삶의 질을 빈도와 백분율, 평균과 표준 편차로 산출하였다. 폐경 여성의 인구사회학적 특성에 따른 삶의 질 차이는 independent t-test로 분석하였으며, 폐경 증상, 부부친밀도, 삶의 질 간 관계는 Pearson correlation coefficients로 분석하였다. 또한 폐경 여성의 삶의 질에 영향을 미치는 요인은 입력 방식의 다중 회귀분석을 시행하였고, Kolmogorov-Smirnov 검정으로 연속변수의 정규성 분포를 확인하였다.

## Results

### 폐경 여성의 폐경 증상, 부부친밀도 및 삶의 질

폐경 증상은 평균 14.85±8.16점(40점 만점)으로 낮은 수준이었으며, 하위요인 중 신체적 증상은 2점 만점에 0.72±0.44점, 정신적 증상은 0.68±0.48점, 성적 증상은 0.85±0.56점이었다. 부부친밀도는 평균 20.81±4.61점(32점 만점)으로 다소 높은 수준이었으며, 4점 만점에 2.60±0.58점이었다. 삶의 질은 평균 86.06±12.49점(130점 만점)으로 다소 높은 수준이었고, 5점 만점에 3.31±0.48점이었다(Table 1).

### 폐경기 호르몬요법 실태

전체 대상자 중 폐경기 호르몬요법 대상자인 97명(50.0%)의 호르몬요법 실태는 다음과 같다. 폐경기 호르몬요법의 평균 시작 연령은 51.96±3.77세였고, 투여 기간은 43.34±40.38개월(범위, 3–192개월)이었다. 호르몬요법 선택 이유는 ‘갱년기 증상’이 86명(64.7%)으로 가장 많았고, ‘골다공증 예방’ 16명(12.0%), ‘의사·간호사의 권유’ 15명(11.3%) 등의 순으로 나타났다. 호르몬요법 중 정기검진 기간은 ‘1년’이 82명(84.5%)으로 가장 많았다. 호르몬요법 효과는 ‘다소 효과적’이 54명(55.7%)으로 가장 많았고, ‘매우 효과적’ 33명(34.0%), ‘보통’ 10명(10.3%) 순으로 나타났다. 호르몬요법 후 완화된 증상은 ‘안면홍조’가 54명(20.2%)으로 가장 많았고, ‘식은땀’ 52명(19.5%), ‘불면증’ 48명(18.0%), ‘질 건조증’ 31명(11.6%), ‘감정 변화’ 25명(9.4%) 등의 순으로 나타났다.

부작용에 대한 설명을 들은 적이 있는 경우가 대부분이었으며, 부작용을 설명한 사람은 ‘의사’가 64명(59.3%)으로 가장 많았고, ‘간호사’는 1명(0.9%)이었다. 부작용 경험이 있는 경우가 33명(34.0%)이었으며, 부작용 증상으로는 ‘유방통’이 13명(32.5%)으로 가장 많았고, ‘부정 출혈’ 12명(30.0%), ‘체중 증가’ 10명(25.0%) 등의 순이었다. 호르몬요법 중단 경험이 있는 경우가 45명(46.4%)이었으며, 중단 이유로는 ‘암에 대한 두려움’ 19명(34.5%), ‘매일 복용이 불편해서’ 9명(16.4%), ‘장기 복용’ 8명(14.5%), ‘부작용이 나타나서’ 7명(12.7%) 등으로 나타났다. 재사용 이유는 ‘갱년기 증상이 심해져서’ 36명(70.6%), ‘노화에 대한 걱정’ 13명(25.5%), ‘부작용이 사라져서’ 2명(3.9%) 순으로 나타났다(Table 2).

### 폐경 여성의 인구사회학적 특성에 따른 삶의 질의 차이

폐경 여성의 삶의 질은 고등학교 졸업에 비해 대학교 졸업 이상에서 유의하게 높았으며(t=–4.20, *p*<.001), 한 달 가정 수입이 300만 원 이하보다 300만 원을 초과한 경우에 유의하게 더 높았다(t=–6.76, *p*<.001). 음주를 하는 경우가 하지 않는 경우에 비해 삶의 질이 유의하게 높았다(t=2.18, *p*=.031). 또한 내과적 질환이 없는 경우가 있는 경우에 비해 삶의 질이 유의하게 높았으며(t=–2.45, *p*=.015), 폐경기 호르몬요법을 하는 경우가 하지 않은 경우에 비해 삶의 질이 유의하게 높았다(t=–9.97, *p*<.001) (Table 3).

폐경 여성의 평균 연령은 55.85±3.79세였으며(범위, 47–65세), 교육수준은 고등학교 졸업 이하가 133명(68.6%), 대학교 졸업 이상이 61명(31.4%)이었다. 한 달 가족 수입은 300만 원 이하가 73명(37.6%)이었고, 300만 원 초과가 121명(62.4%)이었다. 현재 흡연을 하는 경우는 5명(2.6%)이었고, 현재 음주를 하는 경우는 49명(25.3%)이었다. 내과적 질환이 있는 경우는 72명(37.1%)이었으며, 직업이 있는 경우가 79명(40.7%)이었다. 폐경 후 기간은 평균 4.74±3.58년(범위, 1–19년)이었으며, 폐경기 호르몬요법을 하는 경우는 전체 대상자의 절반인 97명(50.0%)이었다(Table 3).

### 폐경 증상, 부부친밀도, 삶의 질 간 관계

폐경 증상, 부부친밀도, 삶의 질 간 관계에서 삶의 질은 폐경 증상과 유의한 음의 상관관계가 있었고(r=–.40, *p*<.001), 부부친밀도와 유의한 양의 상관관계(r=.54, *p*<.001)가 있는 것으로 나타났다(Table 4).

### 폐경 여성의 삶의 질 영향요인

독립변수의 다중공선성 검정에서 공차한계(tolerance)의 범위가 0.73–0.98로 0.1 이상이었고, 분산팽창계수(variance inflation factor)가 1.02–1.36로 10 미만으로 나타나 다중공선성의 문제가 없음을 확인하였다. 또한 잔차의 자기상관성을 검정한 결과 Durbin-Watson 통계량은 1.85으로 2에 가까워 오차항 간에 자기 상관이 없었으며, 잔차의 정규성을 검증한 결과 Kolmogorov-Smirnov 검정에서 z 통계량이 .91 (*p*=.314)로 나타나 잔차의 정규성을 만족하였다.

폐경 여성의 삶의 질 영향요인을 알아보기 위해 인구사회학적 및 폐경기 호르몬요법에서 유의한 차이를 보인 교육(1=대학교 졸업 이상), 한 달 가족 수입(1=300만 원 초과), 내과적 질환(1=없음), 음주(1=예), 현재 폐경기 호르몬요법 여부(1=예)는 더미변수로 처리하였고, 폐경 증상과 부부친밀도와 함께 독립변수로 투입하여 다중 회귀분석을 실시하였다. 그 결과 폐경 여성의 삶의 질 향상 모형은 통계적으로 유의하였고(F=40.98, *p*<.001), 전체 변량에 대한 설명력은 59.2%였다. 폐경 여성의 삶의 질에 영향을 미치는 요인으로는 현재 폐경기 호르몬요법 여부(t=6.32, *p*<.001), 부부친밀도(t=4.94, *p*<.001), 한 달 가족 수입(t=4.78, *p*<.001), 폐경 증상(t=–4.37, *p*<.001) 및 교육수준(t=3.66, *p*<.001)으로 나타났다. 즉 입력한 변수의 상대적 중요도를 평가한 결과, 현재 폐경기 호르몬요법 여부(β=.34)가 가장 중요한 요인이었고, 부부친밀도(β=.26), 한 달 가족 수입(β=.24), 폐경 증상(β=–.21), 교육수준(β=.18) 순으로 나타났다(Table 5).

## Discussion

본 연구에서 폐경 여성의 삶의 질에 영향을 미치는 요인을 확인한 결과, 현재 폐경기 호르몬요법 여부가 가장 큰 영향요인으로 나타났다. 즉, 현재 폐경기 호르몬요법을 할수록 폐경 여성의 삶의 질이 높아짐을 의미한다. Kim과 Lee [24]의 연구에서도 폐경기 호르몬요법을 실시한 여성의 삶의 질이 실시하지 않은 군에 비해 더 높게 나타나 본 연구결과를 뒷받침한다. 폐경기 호르몬요법은 폐경 증상을 완화시키고 스트레스, 성기능, 관계 등을 회복시킴으로써 폐경에 대한 새로운 의미를 형성하게 만든다[8]. 특히 질 건조와 성교통으로 인한 성생활의 불편이 감소되면서 임신 걱정 없는 부부관계를 경험하기도 한다[8]. 또한 호르몬요법을 실시하는 경우 폐경과 노화에 대한 태도가 덜 부정적이며, 사회관계망 간의 갈등이 낮아지기 때문에[27] 삶의 질이 향상될 수 있다. 그러나 Kim 등[5]의 연구에서는 폐경기 호르몬요법군과 비치료군의 삶의 질에 유의한 차이가 없었는데, 이는 농촌 지역 폐경 여성들은 대부분 폐경 증상이 심하지 않으며, 치료군이 비치료군에 비해 혈관운동계 증상에서만 효과가 있는 것으로 나타나 삶의 질에까지 영향을 미치지 못했을 것이라고 하였다. 호르몬요법은 치료시기와 증상에 따른 개별화 치료가 삶의 질을 향상시키는 역할을 할 수도 있지만 증상이나 질환에 따라 약제의 종류와 용량, 효과가 달라질 수 있으며, 혈전증, 관상동맥질환 등의 부작용 위험이 있을 수도 있어 주의가 필요하다[12]. 우리나라의 폐경기 여성들은 호르몬요법에 대한 우려가 높으며, 전문의보다는 대중매체에 의존하여 호르몬요법을 판단하는 경향이 있다[28]. 전문의와 간호사는 호르몬대체요법의 이득과 위험에 따라 올바른 치료 선택을 할 수 있도록 정확한 정보와 전문적인 상담을 제공해야 할 것이다.

다음으로 폐경 여성의 삶의 질에 영향을 미치는 요인은 부부친밀도로 나타났다. 부부친밀도가 높으면 삶의 질이 높아진다는 의미로, 선행연구[10,11]의 결과와도 유사하다. 부부친밀도가 높은 경우 평등한 부부관계에서 원활한 의사소통이 가능하며[11] 건강한 성생활을 유지할 수 있어[17] 삶의 질 또한 높아진 것으로 볼 수 있겠다. 또한 낮은 부부친밀감은 폐경 증상의 증가와 관련성이 있기 때문에[7], 부부친밀도가 감소할수록 폐경 증상이 증가하게 되고, 이로 인해 삶의 질이 낮아질 수 있을 것이라고 본다. 폐경 여성의 삶의 질을 향상시키기 위해 부부친밀감을 강화시키기 위한 프로그램이 개발되어야 하며, 특히 폐경 여성의 부부친밀감은 성기능에 큰 영향을 받으므로, 폐경기 여성과 배우자 모두에게 성기능 향상에 초점을 둔 성 교육 및 성 상담이 이루어져야 할 것이다[17]. 또한 부부 모두 노화를 경험하는 시기이기 때문에[8] 자신이 겪고 있는 신체 및 감정 변화에 치중할 수 있어, 배우자의 신체적·심리적 변화에 관심을 가지고 함께 극복하기 위해 노력해야 할 것이다.

폐경 증상 또한 폐경 여성의 삶의 질에 유의한 영향요인이었으며, 폐경 증상이 심할수록 삶의 질이 낮아지는 것으로 나타났다. 폐경 증상의 위험도는 신체적 건강 관련 삶의 질이 감소할수록 13.38배, 정신적 건강 관련 삶의 질이 감소할수록 5.65배 증가한다는 선행연구[7] 결과와도 비슷한 맥락으로 이해할 수 있겠다. 국외 연구에서도 폐경 증상이 있는 여성이 없는 여성에 비해 삶의 질 점수가 현저히 낮았으며[15], 특히 수면장애와 질 건조 증상이 삶의 질에 가장 큰 영향을 미치는 것으로 보고되었다[16]. 폐경 증상이 심해지면 신체 고통 이외에도 의욕 저하, 우울, 답답함, 스트레스 등 불안정한 정서반응이 나타나고[8], 가족관계나 사회적 지지망으로부터 멀어지며 부부친밀감을 떨어뜨릴 수 있다[7]. 또한 폐경 증상은 만성질환과의 관련성이 높기 때문에[15], 폐경 증상이 심할수록 만성질환이 동반되어 삶의 질 또한 낮아졌을 것으로 생각된다. 폐경 호르몬제의 사용은 혈관운동계 증상, 비뇨생식기 증상, 골다공증에 확실한 효과가 입증되었으므로, 폐경 증상, 위험요소, 치료 목적 등을 고려하여 치료를 실시한다면[13] 폐경 이후 삶의 질을 향상시킬 것이다. 또한 폐경 증상은 만성질환으로 이행될 가능성이 높으며, 시간이 경과할수록 더 심해지기 때문에[8] 폐경 여성 스스로 미리 관리하고 적극적으로 대처해야 할 것이다.

마지막으로 한 달 가정 수입과 교육수준이 폐경 여성의 삶의 질에 영향을 미치는 것으로 나타났다. 한 달 가정 수입이 많고, 교육수준이 높을수록 삶의 질이 높아짐을 의미한다. 폐경 여성의 경우 월 평균 소득과 학력수준이 낮은 경우 의사결정이나 선택 시 여러 환경적 조건을 고려해야 하고 갈등 수준이 높기 때문에[14] 삶의 질에 부정적 영향을 미칠 수 있을 것이라 생각된다. 따라서 경제상태와 학력수준이 낮은 폐경 여성들에게 더 많은 관심을 가지고 이들 계층의 폐경으로 인한 신체 증상이나 정신적 건강문제를 고려한 대책이 마련되어야 할 것이다.

본 연구 대상자의 평균 폐경 연령은 55.85세였으며, 이는 국내 여성의 정상적인 폐경 연령인 48–55세에[1] 비해 다소 높았다. 폐경기 호르몬요법 시작 연령은 평균 51.96세였으며, 선행연구[24,25]에서도 대부분 50대 초반으로 나타났다. 폐경기 호르몬요법을 선택하게 된 이유는 폐경 증상이 가장 많았고, 다음으로 골다공증 예방이었다. Sun 등[14]의 연구에서는 골다공증 예방이 가장 많았고, 다음으로 폐경 증상 완화 등이었다. 호르몬요법은 폐경으로 인한 안면홍조, 땀, 두근거림, 불안과 같은 혈관운동 증상에 일차적 치료제로 사용되고 있으며, 골다공증 예방에 큰 효과가 있음이 보고된 바 있다[9].

호르몬요법의 효과에 대해 90%의 대상자가 효과적이라고 응답하였으며, 기존 연구[25]에서는 75.4%였다. 사용 후 완화된 증상으로는 안면홍조가 가장 많았고, 식은땀, 불면증 순으로 나타났다. 안면홍조, 수면 문제, 발한 등의 증상들은 폐경 후에 유병률이 더 증가한다는 연구 결과[15]를 통해서도 폐경기 호르몬요법에서 우선적으로 기대할 수 있는 효과라 볼 수 있겠다. 대상자의 90%가 호르몬요법 부작용에 대해 들은 적이 있었고, 설명을 한 사람으로 의사가 60%로 가장 많았다. 선행연구[25]에서도 호르몬요법의 복약 지도를 의사로부터 받는 경우가 38.9%였고, 호르몬요법의 정보원으로 의사가 40%로 가장 많았다[29]. Chung 등[28]의 연구에서도 폐경기 호르몬요법의 실시나 지속 여부는 의사들의 조언이 중요한 역할을 하는 것으로 나타났다. 최근 환자의 알 권리와 결정권이 중시되면서 치료에 대한 설명이 더 강화되었으므로, 의사뿐 아니라 의료진도 폐경기 호르몬요법의 이득과 위험에 대한 근거가 명확한 최신 전문지식을 확보하기 위해 노력해야 할 것이다.

호르몬요법 중 부작용 경험은 34%였으며, 증상으로는 유방통이 가장 많았고 부정 출혈, 체중 증가, 부종, 위장장애 순이었으며, 이는 Hong과 Lee [25]의 연구와도 비슷한 결과이다. Chung 등[28]의 연구에서는 질 출혈이나 유방통보다는 체중 증가를 더 많이 호소했다. 폐경 호르몬요법의 부작용으로 질 출혈, 체중 증가, 유방통 등이 흔히 보고되고 있으며[6], 정맥혈전증, 담도 관련 질환의 위험도 증가할 수 있다[6]. 호르몬요법 중단 경험이 있는 경우는 절반 정도였으며, Sun 등[14]의 연구에서는 27.7%로 다소 낮았다. 선행연구[14]에서는 인공폐경 여성이 많았는데, 수술로 인한 저에스트로겐증의 경우 폐경 증상 전 저용량을 예방적으로 사용하기 때문에[21] 중단 경험이 더 낮은 것으로 유추해 볼 수 있다. 호르몬요법 중단 이유는 암에 대한 두려움이 가장 많았는데, Chung 등[28]의 연구에서도 같은 결과를 보였다. 최근 연구들은 장기간의 호르몬요법에도 암의 발생 위험이 증가하지 않았다고 보고하고 있으며, 암에 대한 개인 차이를 고려하여 치료 여부를 결정할 것을 권고한다[6]. 폐경기 호르몬요법 시 적절한 유형, 용량, 투여경로 및 사용기간을 개별적으로 적용하고, 이득과 위험을 주기적으로 재평가한다면 이점을 극대화하고 위험을 최소화시킬 수 있다[30]. 폐경 여성에게 호르몬요법을 위한 개인적인 위험 수준에 대해 평가하고 암에 대한 두려움 없이 치료를 선택할 수 있도록 도와야 할 것이다[6].

종합해볼 때 본 연구에서 현재 폐경기 호르몬요법 여부가 폐경 여성의 삶의 질에 가장 큰 영향요인이었으며, 이는 그 동안 밝혀진 폐경기 호르몬요법의 의학적 치료 효과와 더불어 개인의 총체적인 삶에도 긍정적으로 작용할 수 있음을 의미한다. 대상자의 93%가 부작용에 대한 교육을 받았지만 ‘암 발생에 대한 두려움’이 가장 큰 장애요인이었으며, 다시 복용하게 된 이유가 폐경 증상 때문이라는 점은 폐경기 호르몬요법에 대해 정확한 정보 제공이 필요함을 시사한다. 폐경 여성에게 폐경기 호르몬요법에 대한 올바른 정보를 제공하고 개별 환자에 적합한 치료가 적용된다면, 폐경 증상이 감소하고 삶의 질도 향상될 것이다. 또한 갱년기 증상과 노화에 대한 부부 간 이해와 소통을 통해 부부친밀도를 높이고, 저소득층과 교육수준이 낮은 폐경 여성에게 우선적으로 삶의 질 향상을 위한 적극적 중재가 이루어져야 할 것이다.

본 연구의 제한점은 다음과 같다. 일개 여성전문병원의 외래에 질병이나 검진을 목적으로 방문한 여성을 대상으로 하였기 때문에 연구 결과에 편중 가능성이 있으며, 삶의 질이라는 변수가 신체적, 정신적, 환경적인 다양한 측면에서 장기간 측정이 필요함에도 불구하고 일회성의 설문으로 평가했다는 점이다. 또한 호르몬요법 기간의 편차가 높았다는 점은 연구 결과에 영향을 미쳤을 것이다. 향후 호르몬요법 기간을 구분하여 폐경 여성의 삶의 질을 확인할 필요가 있으며, 대표성이 높은 집단을 포함한 반복 연구를 실시할 것과 본 연구에서 영향요인으로 확인된 변인들을 고려하여 폐경 여성을 위한 삶의 질 향상 프로그램을 개발할 것을 제언한다.

## Figures and Tables

**Table 1.** Menopause symptoms, marital intimacy, and quality of life of post-menopausal women (N=194)

Variable	Possible score range	Mean±SD	Item Mean±SD
Menopausal symptoms	0–40	14.85±8.16	0.74±0.41
Physical	0–22	7.89±4.86	0.72±0.44
Mental	0–8	2.70±1.91	0.68±0.48
Sexual	0–10	4.26±2.79	0.85±0.56
Marital intimacy	8–32	20.81±4.61	2.60±0.58
Quality of life	26–130	86.06±12.49	3.31±0.48

**Table 2.** Characteristics of menopausal hormone therapy use (N=97)

Characteristic	Categories	Value
Age of starting MHT (year)		51.96±3.77
Duration of MHT (month)		43.34±40.38
		(range, 3–192)
Reason for taking MHT^†^	Menopausal symptoms	86 (64.7)
	Prevention of osteoporosis	16 (12.0)
	Health providers’ recommendation	15 (11.3)
	Recommendation of family and friends	12 (9.0)
	Newspapers, magazines	4 (3.0)
Screening timing during MHT	Annually	82 (84.5)
	Every 6 months	10 (10.3)
	Every 3 months	2 (2.1)
	Others	3 (3.1)
Treatment effect of MHT	Very effective	33 (34.0)
	Slight effective	54 (55.7)
	Mood	10 (10.3)
Symptom relief after MHT^†^	Hot flush	54 (20.2)
	Cold sweat	52 (19.5)
	Insomnia	48 (18.0)
	Vaginal dryness	31 (11.6)
	Mood change	25 (9.4)
	Depression	16 (6.0)
	Arthralgia	16 (6.0)
	Dry skin	12 (4.5)
	Headache	8 (3.0)
	Tingling sensation of hands and feet	3 (1.1)
	Urinary incontinence	2 (0.7)
Education about side effects of MHT	Experienced	90 (92.8)
	Did not experience	7 (7.2)
Source of information about the side effects of MHT^†^ (n=90)	Doctor	64 (59.3)
	Media	28 (25.9)
	Friends taking hormone therapy	13 (12.0)
	Pharmacist	2 (1.9)
	Nurse	1 (0.9)
Experience of side effects during MHT	Yes	33 (34.0)
	No	64 (66.0)
	Mammalgia	13 (32.5)
	Metrorrhagia	12 (30.0)
	Weight gain	10 (25.0)
	Edema	2 (5.0)
	Gastrointestinal disturbances	2 (5.0)
	Elevated liver enzyme levels	1 (2.5)
Experience of discontinuing MHT	Yes	45 (46.4)
	No	52 (53.6)
Reason for discontinuing MHT^†^ (n=45)	Fear of cancer	19 (34.5)
	Discomfort experienced in taking medicine every day	9 (16.4)
	Long-term use	8 (14.5)
	Side effects	7 (12.7)
	Reluctant to visit hospitals	5 (9.1)
	No effects	3 (5.5)
	Others	4 (7.3)
Reason for restarting MHT^†^ (n=45)	Aggravated menopausal symptoms	36 (70.6)
	Worries about old age	13 (25.5)
	Disappearance of side effects	2 (3.9)

MHT: Menopausal hormone therapy.

Values are presented as mean±SD or n (%)

† Multiple response.

**Table 3.** Differences in quality of life according to demographic characteristics among post-menopausal women (N=194)

Characteristic	Categories	Value	Quality of life		
			Mean±SD	t	*p*
Age (year)		55.85±3.79			
		(range, 47–65)			
Education	Below high school	133 (68.6)	83.60±12.09	–4.20	<.001
	Above college	61 (31.4)	91.41±11.73		
Monthly income (10,000 Korean won)	≤300	73 (37.6)	79.02±10.50	–6.76	<.001
	<300	121 (62.4)	90.31±11.69		
Smoking	Yes	5 (2.6)	88.80±13.27	0.61	.621
	No	189 (97.4)	85.98±12.50		
Drinking	Yes	49 (25.3)	89.38±11.57	2.18	.031
	No	145 (74.7)	84.94±12.63		
Medical disease	Yes	72 (37.1)	83.23±10.89	–2.45	.015
	No	122 (62.9)	87.73±13.11		
Employment	Yes	79 (40.7)	86.73±11.16	0.62	.536
	No	115 (59.3)	85.60±13.36		
Time passed since menopause (year)		4.74±3.58 (range, 1–19)			
Current MHT	Yes	97 (50.0)	93.34±10.28	–9.97	<.001
	No	97 (50.0)	78.78±10.05		

MHT: Menopausal hormone therapy.

Values are presented as mean±SD or n (%)

**Table 4.** Relationships among age, duration after menopause, menopausal symptoms, marital intimacy, and quality of life (N=194)

Variable	r (*p*)			
	Age	Duration after menopause	Menopausal symptoms	Marital intimacy
Duration after menopause	.68 (<.001)	1		
Menopausal symptoms	–.20 (.005)	–.15 (.038)	1	
Marital intimacy	–.01 (.833)	.00 (.918)	–.22 (.002)	1
Quality of life	.08 (.224)	.06 (.393)	–.40 (<.001)	.54 (<.001)

**Table 5.** Factors Influencing quality of life among post-menopausal women (N=194)

Variable	B	SE	β	t	*p*
(Constant)	47.59	5.08		9.36	<.001
Education ^†^	4.81	1.32	.18	3.66	<.001
Monthly family income ^†^	6.06	1.26	.24	4.78	<.001
Presence of medical disease ^†^	2.34	1.20	.09	1.95	.053
Drinking ^†^	2.25	1.36	.08	1.66	.099
Current MHT ^†^	8.45	1.34	.34	6.32	<.001
Menopausal symptoms	–0.33	0.08	–.21	–4.37	<.001
Marital intimacy	0.70	0.14	.26	4.94	<.001
	Adjusted R^2^=59.2%, F=40.98, *p*<.001				

† Dummy variable references were education (up to high school), monthly family income (≤ 3 million Korean won), presence of medical disease (yes), drinking (no), and menopausal hormone therapy (no).
